# Why equating all evidence searches to systematic reviews defies their role in information seeking

**DOI:** 10.5195/jmla.2019.707

**Published:** 2019-10-01

**Authors:** Zachary E. Fox, Annette M. Williams, Mallory N. Blasingame, Taneya Y. Koonce, Sheila V. Kusnoor, Jing Su, Patricia Lee, Marcia I. Epelbaum, Helen M. Naylor, Spencer J. DesAutels, Elizabeth T. Frakes, Nunzia Bettinsoli Giuse

**Affiliations:** Associate Director for Information Services, Center for Knowledge Management, Strategy and Innovation, Vanderbilt University Medical Center, Nashville, TN, Zachary.e.fox@vumc.org; Senior Information Scientist, Center for Knowledge Management, Strategy and Innovation, Vanderbilt University Medical Center, Nashville, TN, annette.williams@vumc.org; Information Scientist, Center for Knowledge Management, Strategy and Innovation, Vanderbilt University Medical Center, Nashville, TN, mallory.blasingame@vumc.org; Associate Director for Research, Center for Knowledge Management, Strategy and Innovation, Vanderbilt University Medical Center, Nashville, TN, taneya.koonce@vumc.org; Senior Research Information Scientist, Center for Knowledge Management, Strategy and Innovation, Vanderbilt University Medical Center, Nashville, TN, sheila.v.kusnoor@vumc.org; Information Scientist, Center for Knowledge Management, Strategy and Innovation, Vanderbilt University Medical Center, Nashville, TN, jing.su@vumc.org; Senior Information Scientist, Center for Knowledge Management, Strategy and Innovation, Vanderbilt University Medical Center, Nashville, TN, patty.lee@vumc.org; Senior Information Scientist, Center for Knowledge Management, Strategy and Innovation, Vanderbilt University Medical Center, Nashville, TN, marcia.epelbaum@vumc.org; Senior Research Information Scientist, Center for Knowledge Management, Strategy and Innovation, Vanderbilt University Medical Center, Nashville, TN, helen.naylor@vumc.org; Information Scientist, Center for Knowledge Management, Strategy and Innovation, Vanderbilt University Medical Center, Nashville, TN, spencer.desautels@vumc.org; Information Scientist, Center for Knowledge Management, Strategy and Innovation, Vanderbilt University Medical Center, Nashville, TN, elizabeth.frakes@vumc.org; Vice President for Knowledge Management; Professor, Department of Biomedical Informatics; Professor, Department of Medicine; and Director, Center for Knowledge Management, Strategy and Innovation, Vanderbilt University Medical Center, Nashville, TN, nunzia.giuse@vanderbilt.edu

## Abstract

All too often the quality and rigor of topic investigations is inaccurately conveyed to information professionals, resulting in a mischaracterization of the research, which, if left unchecked and published, may in turn mislead potential readers. Accurately understanding and categorizing the types of topic investigation searches that are requested of information professionals is critical to both meeting requestors’ needs and reflecting their intended methodological approaches. Information professionals’ expertise can be an invaluable resource to guide users through the investigative and publication process.

Systematic reviews have long played an important role in the evidence hierarchy [1], yet there continues to be confusion regarding what constitutes a true systematic review [2]. In spite of this misunderstanding, systematic reviews figure largely in both the information science and biomedical literature, to the point of the National Library of Medicine adding “systematic reviews” as a new publication type (allowing greater search precision compared with the previous search strategy filter) and “Systematic Reviews as Topic” to the Medical Subject Headings (MeSH) vocabulary for 2019 [3].

With the ongoing expansion of the body of systematic reviews in the literature comes the expansion of articles *labeled* as “systematic reviews” that potentially do not meet the rigorous criteria of systematic reviews [2]. Such mislabeling could result from many causative factors, such as a lack of education and training on what constitutes a systematic review and the incorrect perception that articles using such a label employ comprehensive and complete searches. As a result, a cycle of misconceptions about systematic reviews risks being perpetuated. In our role as information professionals, it is our responsibility to actively clarify the indications for and differences between systematic reviews, “systematic-like” reviews, comprehensive literature reviews, and patient-specific precision inquiries as we proactively engage in training our users and colleagues alike.

A deep and clear-cut understanding of the nature of *true* systematic reviews continues to elude the research community, despite guidance from the Preferred Reporting Items for Systematic Reviews and Meta-Analyses (PRISMA) Statement [4], Agency for Healthcare Research and Quality (AHRQ) [5], Cochrane Collaboration [6], and National Academies of Medicine (formerly, Institute of Medicine) [7]. Given this easy access to trusted, authoritative guides on the standards required of systematic reviews, we need to ask ourselves the question of *why* we continue to see the term “systematic review” improperly applied in published studies to describe less rigorous types of information inquiry [8], despite our profession being regularly engaged in systematic review collaborations, workshops, and professional development activities?

Unfortunately, the root cause can likely be traced to a lack of accurate understanding of the aims and goals of how the search inquiry results will ultimately be used. Physicians, researchers, and even information professionals tend to deem studies or design methods loosely resembling those used in the systematic review process as actual systematic reviews, despite incompleteness and lack of adherence to all the parameters necessary to be a true systematic review. Although these errors are often unintentional, the continued volume of erroneously labeled information search inquiries devalues the term “systematic review,” which is intended to denote independent, unbiased, and objective assessment of evidence and includes rigorous evaluation of the strength of study results and analysis of study bias, painstakingly described for methodological transparency and reproducibility [5, 9].

Staff at the Center for Knowledge Management (CKM) are too often erroneously asked for assistance in conducting “systematic reviews.” As a result, we have armed ourselves with an approach to guide the requester to the type of searching and information retrieval support that is most suited to their needs, while also continuing to educate our professionals on the proper use and labeling of the different types of searches. So, we ask: Does the user really want to conduct a true *systematic review* and adhere to all criteria established by the aforementioned authoritative guides? Or is the user seeking what CKM has coined a “*systematic-like” review*, a review that incorporates some features of a systematic review without adhering to all the required components [10]? Or do they want what Cochrane and AHRQ have come to define as a *rapid review*, done for topics that are still emerging and for which little has been published [11–13]? Or is it possible that the user’s needs can be met with another method of inquiry, such as a *comprehensive literature review* or a *patient-specific precision investigation* with or without content filtering of the information retrieved? It is very telling how much confusion can be eliminated when this simple process is followed; thus showing the need for us, in our role as educators and information providers, to become better versed in the labeling of searches as we inquire and collect data on the type of information need our users are seeking.

Per guidance from multiple organizations, systematic reviews must meet specific requirements [5–7, 9]. Systematic reviews must adhere to a structured, predefined protocol that governs the entire review process: from the formulation of key questions to the writing of the final manuscript. Once a protocol has been established, information professionals perform well-documented, exhaustive searches of relevant sources for appropriate materials. Following comprehensive searching, all retrieved study articles are screened independently by at least two individuals for inclusion in the analysis based on their relevance to the key questions and their ability to meet predetermined inclusion and exclusion criteria. The first screening process is of titles and abstracts only; the second screening is at the full-text level. Data are extracted from full-text articles that meet the eligibility criteria. These studies are also assessed for bias and the strength of the evidence, and the findings are presented in the form of a finished manuscript. The entire systematic review process can take upward of eighteen months to complete [14].

On the other hand, “systematic-like” reviews allow investigators flexibility to select from the standard list of systematic review components and achieve a much shorter turnaround time than that required of true systematic reviews. For instance, a small group of physicians may believe they want to conduct a systematic review but may not be interested in devising a protocol or have the time to conduct risk of bias assessments of selected studies. Lacking such elements precludes the resulting manuscript from being categorized as a true systematic review; however, the application of parts of the systematic review methodology should be recognized.

We are not alone in this attempt to categorize studies falling short of the gold standard for systematic reviews. Another form of categorization that draws from systematic reviews is the “rapid review,” which has arisen in the last ten years or so to more accurately convey the idea that a quickly performed inquiry is somewhat rigorous without being a full-fledged systematic review [15]. These “rapid reviews” are utilized by the AHRQ and Cochrane for topics that are emerging or for which there is an insufficient body of publications to conduct a formal systematic review [11, 13]. Rapid reviews offer a viable option for investigators who are seeking to publish on a short turnaround as they can be completed in less than eight weeks [16]. Although relatively new, these rapid reviews are gaining traction, and their methodologies are still evolving.

Another area where all too frequently users misappropriate the term “systematic review” is in lieu of a comprehensive literature search. The investigator knows that they want a very thorough expert search and review of the biomedical literature, but they understand “systematic” to mean “planned, organized, and methodical.” Comprehensive literature searches that CKM conducts involve an extensive expert review of the relevant published and grey literature; use of more than one database, with or without filtering and synthesis of the resulting articles; and an unbiased presentation of the literature around a given topic [17]. These comprehensive literature searches can be performed in a fraction of the time required for a systematic review and are immediately valuable to the clinician or researcher to answer questions of interest without developing a protocol, adjudicating articles, formally assessing risk of bias, or performing other steps of the systematic review process.

The inherent value of these types of reviews stems from their comprehensiveness. For instance, researchers can take solace in an information professional saying, “There is no answer,” knowing that the topic has been exhaustively explored. Clinicians who may be seeking answers to clinical questions, without wanting to disclose patient-specific information, can rest assured knowing that all the relevant evidence has been considered and the most rigorous studies are being selected. Answering these questions still requires the same level of careful “systematic searching” without necessitating a full-fledged systematic review. These searches address questions such as what the literature says about treating a certain condition, whether a research question has been sufficiently investigated by others, what a standard work-up for a patient presenting to a particular service in the clinical environment is, or whether a clinician has taken the best course of action. If the 2,400 most recent complex questions in our internal database are any indication, this type of search can take an average of 8 hours to complete.

Patient-specific precision investigation allows the highest level of personalization in searching. These are questions pertaining to a single patient that come directly from clinicians and researchers and may be submitted through the medical record, morning reports, or patient conferences such as tumor boards [17–20]. Context of the patient case, such as a complex medical history and multiple comorbidities, are considered when evaluating the applicability of evidence to the clinical query. Questions falling into this category are often best handled by information professionals with deep medical content knowledge gained through tireless research, professional development, and time spent absorbing the latest news and advancements in the field to further one’s understanding of a topic. This type of inquiry can still be described as systematic in its nature, while not falling into the category of a systematic review.

Fixing the cycle of mis-categorization requires more than educating a set of researchers and physicians on the differences in topic investigations: it involves a concerted effort on the part of information professionals to educate ourselves on the key differences in the types of information inquiry asked of us and then actively working to stop perpetuating the misuse of terms by acquiescing to pressure from collaborators. Information professionals who have been involved in the development of true systematic reviews can attest that searching the biomedical literature in a systematic fashion only scratches the surface of what is entailed in genuine systematic reviews, which to be complete also require the active collaboration of content experts for data evaluation and final construct. The genuine impact of all other types of searches remains unquestioned when their role and appropriateness of use is clearly understood and properly applied. Teaching users how to properly label their requests based on their needs will greatly improve the significance of our role as coaches, educators, and information providers in the communities we are charged to inform.

**Zachary E. Fox, MSIS**, Zachary.e.fox@vumc.org, Associate Director for Information Services, Center for Knowledge Management, Strategy and Innovation, Vanderbilt University Medical Center, Nashville, TN

**Annette M. Williams, MLS**, annette.williams@vumc.org, Senior Information Scientist, Center for Knowledge Management, Strategy and Innovation, Vanderbilt University Medical Center, Nashville, TN

**Mallory N. Blasingame, MA**, mallory.blasingame@vumc.org, Information Scientist, Center for Knowledge Management, Strategy and Innovation, Vanderbilt University Medical Center, Nashville, TN

**Taneya Y. Koonce, MSLS, MPH**, taneya.koonce@vumc.org, Associate Director for Research, Center for Knowledge Management, Strategy and Innovation, Vanderbilt University Medical Center, Nashville, TN

**Sheila V. Kusnoor, PhD**, sheila.v.kusnoor@vumc.org, Senior Research Information Scientist, Center for Knowledge Management, Strategy and Innovation, Vanderbilt University Medical Center, Nashville, TN

**Jing Su, MD, MS**, jing.su@vumc.org, Information Scientist, Center for Knowledge Management, Strategy and Innovation, Vanderbilt University Medical Center, Nashville, TN

**Patricia Lee, MLS**, patty.lee@vumc.org, Senior Information Scientist, Center for Knowledge Management, Strategy and Innovation, Vanderbilt University Medical Center, Nashville, TN

**Marcia I. Epelbaum, MA**, marcia.epelbaum@vumc.org, Senior Information Scientist, Center for Knowledge Management, Strategy and Innovation, Vanderbilt University Medical Center, Nashville, TN

**Helen M. Naylor, MS**, helen.naylor@vumc.org, Senior Research Information Scientist, Center for Knowledge Management, Strategy and Innovation, Vanderbilt University Medical Center, Nashville, TN

**Spencer J. DesAutels, MLIS**, spencer.desautels@vumc.org, Information Scientist, Center for Knowledge Management, Strategy and Innovation, Vanderbilt University Medical Center, Nashville, TN

**Elizabeth T. Frakes, MSIS**, elizabeth.frakes@vumc.org, Information Scientist, Center for Knowledge Management, Strategy and Innovation, Vanderbilt University Medical Center, Nashville, TN

**Nunzia Bettinsoli Giuse, MD, MLS, FACMI, FMLA**, nunzia.giuse@vanderbilt.edu, Vice President for Knowledge Management; Professor, Department of Biomedical Informatics; Professor, Department of Medicine; and Director, Center for Knowledge Management, Strategy and Innovation, Vanderbilt University Medical Center, Nashville, TN
